# Economic development trends in the EU tourism industry. Towards the digitalization process and sustainability

**DOI:** 10.1007/s11135-020-01056-9

**Published:** 2020-10-26

**Authors:** Beata Zofia Filipiak, Marek Dylewski, Marcin Kalinowski

**Affiliations:** 1 Institute of Economics and Finance, University of Szczecin, Szczecin, Poland; 2 WSB University in Poznan, Poznan, Poland; 3 WSB University in Gdansk, Gdansk, Poland

**Keywords:** Sustainability, Economic and financial development, Digitalization, Tourism, G 30, L 83, L89

## Abstract

From an economic viewpoint, tourism is heralded as bringing income to local communities. From an ecological standpoint, tourism poses a threat to environments. Sustainable tourism should leave a minimum negative impact on the places visited and preferably have rather positive impact on society. The digitization of the tourism economy is conducive to increasing the efficiency of enterprises operations, but also have positive impact on consumers. The objectives of the study are: to seek an answer to the question whether there is a relationship between the development of the tourism industry and GDP growth. Based on it there are two specific questions: What is a relationship between the level of development of digitization (e-commerce) and the development of the tourism industry and what is a relationship between the development of the tourism industry and sustainability factors? The originality of our research results among others results from three groups of variables use in the analysis (ICT group, SDG group and E&T group). Our research explores the factors affecting the tourism industry and relations of the digitization of tourism economy, sustainability and economy growth.

## Introduction

Digital transformation, as the integration of digital technology into the tourism industry (which includes all businesses that directly provide goods or services to facilitate business, pleasure and leisure activities away from the home environment), results in fundamental changes in the way the world does business, communicates and develops on national and international levels. Customer habits are also changing along with breakthroughs in technology (Hojeghan and Esfangareh 2011) ( Fereidouni and Kawa 2019). Digitization offers many new opportunities that can be exploited by providers in the tourism industry. At the same time, competition is intensifying and companies have to keep pace with digitization in order to remain on the same level.

The development of ICT technology over the last decades has dramatically affected the tourism sector, insofar as the accelerated connection of technologies and tourism in recent years has led to necessary changes in the understanding of the nature of tourism, and requires continuous research and analysis of how digitization affects the economic growth of enterprises in the tourism industry. Research most often targets information technology in tourism and has been almost exclusively focused on the benefits and the applications of technology (Del and Baggio 2015) (Huang et al. 2017) ( Fereidouni and Kawa 2019), and much less frequently on the drawbacks (Gretzel 2011). There are also studies in the literature on the economic effects of digitization. A question which remains unanswered, and which scientists are still looking for answers to, is: “Can digitization be viewed as the motor of transformation for the tourism industry in the age of the internet economy?” (Bauer et al. 2008).

Studies show that digitization offers promising potential in the tourism industry. All business processes occurring in the creation of development in the tourism industry are affected (Ralph and Searby 2004) (Ighalo 2014). In addition to the digital transformation of processes, digitization offers opportunities for new business models in the tourism industry (Souto 2015).

In the face of crises, changes on the market, and especially when it comes to specific environmental factors (e.g. the Greek crisis), the realities of the tourism industry are changing. In most cases, they also exhibit sharply redefined business models, which are highly disruptive to traditional paradigms (Cuesta et al. 2015); (Rayna and Striukova 2016). The recent global financial crisis has demonstrated how important the stability of the economy and financial system is in the modern world. The course of the crisis and its negative consequences in both the regulatory and real spheres have led to the verification (reevaluation) of many, seemingly solid, views on the functioning of economies. The EU financial perspective for 2014–2020 notably takes into account environmental factors, in particular compliance with the idea of sustainability. The tourism industry, which is one of the world’s fastest-growing industries, is now trying to move towards sustainable and responsible practices. Perhaps we should write that this industry was the fastest growing, because it is known that the COVID-19 pandemic caused rapid changes in its development ( Welford et al. 1999) (Kişi 2019). The impact of COVID-19 on the economy is significant, not least in the tourism industry. Factors such as the COVID-19 pandemic, the disruption of ecological balance due to global warming, the loss of social values, and the failure to preserve natural, historical, social, and cultural assets make sustainable tourism a necessity (Kişi 2019).

In view of the fact that sustainability generally involves several separate issues such as the protection of ecological systems, intergenerational equity and the efficiency of resource use (Heal 1998), the valuation of environmental assets and the recognition of constraints implied by the dynamics of environmental systems (Jones and Dowling 2004) (Matthes 2007a, b) (Ziolo et al. 2019), then it also implies the need to look at externalities and their impact on tourism. The natural environment implies the development of a tourist economy. The basis for determining the types of externalities is the consideration of axioms which define sustainability. It is about considering a number of sustainability factors that will determine the development of the tourism economy^1^.

In the report “Our Common Future”, four domains of sustainability are indicated: economy, ecology, politics, and culture (WCED 1987). It should be remembered that sustainable development is a guarantee of a good quality of life and is a way of organizing the social and economic life of a human being (Paul and Liam 2016). However, decisions made by public authorities in the area of striving for sustainable development imply the need to take sustainability factors into account in terms of the policy of enterprises in the tourism sector (WTO 2020).

The study contributes to existing research, covers the gap in the existing literature, and provides a complex theoretical framework for defining and understanding the problem of economic growth in the tourism sector and its role in the contemporary economy from the perspective of achieving sustainable development goals (United Nations 2015).

The paper aims to contribute to the body of knowledge of factors affecting the tourism industry, especially providing a new general theory pertaining to the influence of sustainability and digitalization on the tourism industry. We also want to broaden the abovementioned question as follows: Can digitization, taking into account the factors of sustainability, be viewed as the motor of transformation for the tourism industry in the age of the internet economy?

The paper is organized as follows: an introduction has been presented in Sect. 1. Section 2 discusses the literature review. Section 3 presents the data, the variable description and methodological framework. Finally, Sect. 4 provides the empirical results and conclusions.

## Literature review

In view of the fact that sustainability generally involves several separate issues such as the protection of ecological systems, intergenerational equity and the efficiency of resource use (Heal 1998), the valuation of environmental assets and the recognition of constraints implied by use of the environment (Matthes 2007a, b) (Ziolo et al. 2019), then it also implies the need to look at issues related to tourism development, the role of digitization and its impact on economic growth.

Research interest in factors affecting the tourism industry, especially providing a new general theory pertaining to the influence of sustainability and digitalization on the tourism industry, has increased recently.

The tourism sector has undergone a remarkable change due to advances in digitization processes and transformation as a result of these advances. Digitization in tourism has implications for farming processes and has impacted the economic efficiency of the tourism industry. Research has indicated the presence of factors that influence both consumer and directly economic growth. In the course of our research, we will attempt to find a number of indications regarding the economic benefits associated with the evolution and use of digitization in tourism, in terms of opportunities and access to supply and information. However, there is a lack of research on the impact of digitization on the development of sustainable tourism. In addition to directional studies, numerous researchers point to a number of factors affecting the tourism industry, in particular economic growth factors. The literature on the subject shows trends in the evolution of the impact of the different factors on the tourism economy. Studies are evolving, as in turn are approaches to this topic, deepening existing research and exploring new trends. Pursuant to the literature review, there are many different relationships and possibilities inherent in analyzing the factors affecting the tourism industry. The directional evolution of the research is summarized in Table 1.

**Table 1:** Research directions on the effects of digitization on the tourism economy (with particular emphasis on factor analysis). Source: own study

Research directions	Author	Type of factors or relations which were analyzed
The relationship between digitization and various factors affecting the tourism economy	(Gretzel et al. 2000) (Dellarocas 2003)	Increased sales
	(Gretzel et al. 2000) (Tiefenbeck et al. 2016) (Mistliis et al. 2014)	Classic booking
	(Azevedo and Weyl 2016) (PWC 2015)	Sharing economy
	(Alford 2005) (Buhalis and O´Connor 2005) (Sundararajan 2014)	Process costs
	(Ralph and Searby 2004) (Buhalis and Amaranggana 2014) (Harasymowicz 2015)	Personalized offers
	(Bennet 2012) (Leung et al. 2012) (Hudson and Thal 2013)	Social media
	(Xiang and Gretzel 2010) (Ighalo 2014) (Book et al. 2016)	Customer reviews
	(Huang et al. 2016) (Kim and Hall 2019)	Virtual reality
Defining four criteria for tourism which can stimulate many changes mainly related to economic growth: employment sector, penetration rate, technology and the value factor	(Buhalis and Law 2008)	Relationship between economic growth and the employment sector, penetration rate, technology and the value factor
Research on policy factors: the digitization of tourism as a unique industrial policy goal; the failure to face this challenge could have wide-ranging economic consequences	(Stiakakis and Georgiadis 2011) (Gruber 2017) (Okhimenko et al. 2019)	Influence of policy factors
Stability of systems and data: Big Data could potentially also be used for internal risk management and outside monitoring of financial services and institutions, and thus make supervision more efficient	(Adamczewski 2016)((Dorofeyev et al. 2018)	Impact of system and data stability
The impact of financial services on the development of the tourism economy by means of digitization	(Keller B. 2015) (Pshenichnikov 2017) (Afonasova et al. 2019)	Financial services
Research on digitization in the tourism economy shows that, despite the flexibility of operation, efficiency improves with rapid introduction of changes and the tendency to have a cheap structure is strengthened A quick redefinition of business models can lead to activity which is highly disruptive to traditional paradigms	(Cuesta et al. 2015) (Rayna and Striukova 2016)	The impact of new technologies on business models
Research on the impact of digitization on the productivity of the tourism economy. In particular, researchers sought the answer raising the question of a possible productivity paradox in the digital economy was sought and they looking for the answer whether productivity in industrialized countries now confronts an apparent decline	(Gorelova 2016) (Watanabe et al. 2018)	Productivity in industrialized countries
Research on various measurement methods and factors affecting the level of GDP and the impact of digitization on measuring international transactions and assets, as well as the scope of works and services	(Cockayne 2016) (Ahmad and Schreye 2016) (Watkins et al. 2018)	GDP and impact for measuring international transactions and assets, as well as the scope of works and services
Research on sustainable tourism. The need to reconcile economic growth and sustainable development also has an ethical dimension in tourism	(Sustainable Tourism…, 2017) (Sofronov 2017)	Research on factors such as GHG, energy, water in combination with improving the quality of tourism jobs, preserving natural and cultural resources, limiting negative impacts at tourist destinations, including the use of natural resources and waste production;-promoting the wellbeing of the local community;-reducing the seasonality of demand;-limiting the environmental impact of tourism-related transport
Research on the thesis that reducing human intervention and making everything connected increases efficiency	(Mostafa et al. 2019)	Limiting human intervention
Implementing IT in the tourism economy and industry can lead to economic and social transformation	(Stiakakis and Georgiadis 2011) (Graham et al. 2017) (Haseeb et al. 2019)	Influence of IT on economic and social transformation
Impact of sustainable business performance on the tourism economy	(Haseeb et al. 2019)	Sustainable business performance

Although digitalization is a rapidly developing sphere of national interest—especially when it comes to the tourism economy—and has both advantages and disadvantages, scientists differ when it comes to the direction of their views. The emergence of new technologies is first to indicate a change in the economic systems, not to mention their reputation of providing tourist services as the drivers of economic development. The evolution of research trends in the tourism economy has shown that there are changes in the search for factors affecting economic growth in the tourism economy. Along with the development of society, progressive industrialization, environmental degradation and independent factors that rapidly affect the tourism economy (e.g. factors causing rapid changes such as COVID-19), there is a real need to study the impact of various factors on economic growth in tourism in changeable social and economic conditions.

The fact that there is a real need (taking into account the general trend of sustainable development and a sense of social responsibility) for the development of sustainable tourism (Welford et al. 1999) (Haseeb et al. 2019) should be taken into account; however, the significant impact of digitization on economic growth in this industry is observed. Two main directions of research on factors influencing the development of the tourism industry are indicated (digitalization and research on economic growth—Table 1). There are no binding arrangements regarding the links between sustainability and digitization and GDP research in the tourism economy. The existing research gap is the combination of digitization, sustainable development and corporate social responsibility. Therefore, in our opinion, there is a need to determine which digital and environmental factors have a significant impact on economic growth in the tourism sector.

Through digitization, many processes in tourism companies have become more effective, and thus more cost-efficient. This results in a large potential sales volume because the use of the Internet makes the transition and distribution of information quicker, better, and cheaper regardless of geographical and time limitations.

Consumers and have faster (more direct) access to offers, knowledge and conditions as well as protection of their interests. They can become familiar with the specifics of a place and assess whether it meets their sustainability requirements (Haseeb et al. 2019). Digitization allows one to assess ESG risk factors (Ziolo et al. 2019) and incorporate them into the decision-making process. Externalities may be positive (benefits) or negative (costs) for enterprises. We can also consider them in the form of the provision of services and the effects of consumption. Although the discussion on externalities has been around for a long time, the concept is still controversial. From the point of view of sustainable development, the external effects will be associated with three basic pillars: the environmental pillar, the social pillar and the economic pillar (Zhao et al. 2018) (Ziolo et al. 2019). Corporate social responsibility (CSR) is a concept with constantly increasing importance for tourism businesses and their stakeholders. The environmental, social and governance (ESG) dimensions of CSR performance may contribute to economic performance of tourism businesses. Environmental, social, and governance issues are important for stakeholders and for the customers. Tourism businesses use CSR as a strategic tool to create favorable stakeholder and customers perceptions.

**Fig. 1:**
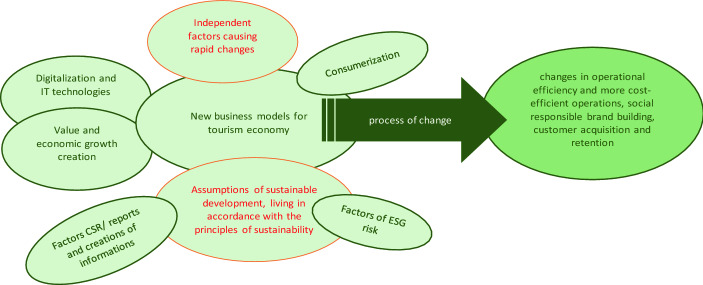
The concept of connections between digitization, sustainable development and changes in the efficiency of the tourism economy. Source: own elaboration

CSR can ensure that customers perceptions are not influenced negatively by activities which they might deem unsustainable (Palazzo and Richter 2005; Yoon et al. 2006; Sila and Cek 2017). The literature on the subject indicates that other stakeholders are also demanding more and more CSR information (O’Dwyer et al. 2005). In the literature review, CSR performance is measured by the ESG performance scores. (Richardson 2009; Cuesta and Valor 2013; Sila and Cek 2017). It is also indicated that information on ESG factors may be disclosed in the CSR reports of tourism enterprises. This information may sometimes be biased, called “greenwashing” (Galbreath 2013; Sila and Cek 2017), where companies exaggerate the level of their CSR practices to create a more positive corporate image to their stakeholders and especially customers. The sphere of ESG and CSR is significantly linked by the digitization process, which enables the collection, provision of information and shaping customer attitudes and decisions of other stakeholders. Figure 1 presents the idea of connections between digitization and sustainable development, taking into account the consumers of the tourist industry.

When assessing the contribution of tourism, it can be stated that this sector plays a key role in the implementation of sustainable development goals (WTTC, WTO and the Earth Council 1995) (UN General Assembly 2015). The power of digital information for customers of tourism enterprises, associated with the digital transformation of business models, is creating a new ecosystem and a new way of doing business. The “blue ocean theory” is once again being proved, because the speed of technology is creating a higher level of pressure for the existence of movements and the transformation of companies to find new “Blue Oceans”. This means that tourism companies are transforming their ways of doing business, not only in terms of internal agility and efficiency processes, but also when it comes to external interactions with their existing and prospective customers (Dellarocas 2003) (Zimmermann et al. 2016) (Ribeiro and Florentino 2016).

A growing number of studies indicate expectations regarding the improvement in the social, environmental and economic results of enterprises which use sustainability ideas in their business models. This evidence, and the fact that we may observe a systematic increase in the costs of social and environmental damage as a result of negative externalities, indicates the need for a strong pressure to create sustainable value, especially in the tourism economy. From this point of view, there is a great deal of space for sustainability as a new element of economic growth in the tourism economy. The specific objectives of the study are:

- to seek an answer to the question of whether the tourist economy has an impact on sustainable economic growth,
- to investigate whether the digitization of the tourism economy affects the stability of revenues and functioning of business tourism,
- determining if (and which) digital and environmental factors have a significant impact on economic growth in the tourism sector.

## Methodology and indicators

### Statistical materials

Considering that the basic goal of our research was to answer the question of whether digitization, taking sustainability factors into account, can be seen as a motor for the transformation of the tourism industry in the age of the internet economy, we were obliged to select a representative set of variables to study. The empirical analyses presented in this paper take the countries of the European Union into account and are based on three groups of data (Table 2) related to: economic and tourism factors (E&T), ICT factors (ICT) and Sustainable Development Goals (SDGs).

**Table 2:** Explained variables included in research

	Indicators		Justification for the Choice
		Data base	Utilization of the variable in research (selected examples):
E&T group	National accounts aggregates by industry (Economic): Value added, gross Operating surplus and mixed income	European Commission; Eurostat Data Base: Economy and Finance, National Accounts (ESA 2010)	The economic effects of tourism, business development and in the context of sustainable and healthy tourism development to measure the tourism industry’s contribution and leakages (Boz 2011); tourism as a source of economic growth (Dwyer et al. 2009); digital transformation and its influence on GDP (Mićić 2017); the impact of digital development on the tourism industry as a function of GDP (Watkins et al. 2018)
	Annual detailed enterprise statistics for services (Tourism) Enterprises–number Turnover or gross premiums written Number of establishments, bedrooms and bed-places Arrivals at tourist accommodation establishments	European Commission; Eurostat Data Base: Industry Trade and Services, Tourism	Variables considered in the context of welfare of individuals, household income, employment, private revenue etc. and also an increase in government revenue (FaladeObalade and Dubey 2014); research on the impact of tourism infrastructure on the size of the tourism economy (Mesjasz-Lech 2017); studies on the empirical relationship between international tourism and the adoption of digital technologies (Lopez-Cordova 2020); the relationship between the number of companies and digitization and the number of enterprises and economic growth (Trașcă et al. 2019)
ICT group	Enterprises with internet access	European Commission; Eurostat Data Base: Science Technology Digital Society	Survey on the effects of the digital economy on the tourism industry through Internet and Web technologies (Hojeghan and Esfangareh 2011); (Adamczewski 2016)
	Websites and functionalities: Enterprises where the website had online ordering, reservation or booking and at least one of: webacc, webctm, webot or webper		Identifying and developing ICT tools for tourism innovation (Mićić 2017); the use of diverse ICTs has a positive effect on market share and economic growth (López and Aramendia-Muneta 2013); ICT as an innovative approach to managing sustainable tourism development (Ali and Frew 2014)
	E-commerce sales A minimum of 1% of turnover generated from online sales	European Commission; Eurostat Data Base: Sustainable development indicators	The impact of e-commerce on development (Ying 2017); the impact of e-commerce on profitability and sustainability (Elhaj and Barakeh 2015) (Hojeghan and Esfangareh 2011);
SDGs group	Promote sustained, inclusive and sustainable economic growth, full and productive employment and decent work for all: Real GDP per capita (economic and sustainability)		It is recognized that for the tourism economy, and especially for safe and active leisure, factors such as inland water bathing sites with excellent water quality, peaceful and inclusive societies without conflicts or incidents affecting security, accountable and inclusive institutions at all levels and climate action are important. The European Commission, through Eurostat, is analyzing the impact of these factors in various cross-sections of sustainability. They were also studied in the context of the tourist economy (Marttunen et al. 2019) (Frone and Frone 2013) (Kovari and Zimanyi 2011) (Brondoni 2016) (Eijgelaar et al. 2010)
	Ensure the availability and sustainable management of water and sanitation for all: Inland water bathing sites with excellent water quality		
	Promote peaceful and inclusive societies for sustainable development, provide access to justice for all and build effective, accountable and inclusive institutions at all levels: General government total expenditure on law courts		
	Climate action: Greenhouse gas emissions		

Source: own elaboration

The first part of the study begins with a critical analysis of the field literature, highlighting both quantitative and qualitative studies on the impact of digitalization on tourism and economic growth. In the literature analysis, we looked at the relationship between digitalization and its impact on the business performance of tourism industry enterprises, as well as the relationship between the tourism economy and sustainability. The critical analysis of the field literature has led us to determine variables that affect the development of the tourism industry and GDP growth, and the level of development of digitization and the development of the tourism industry. We include these variables in Table 2. Furthermore, we make use of an empirical analysis of the E&T group, the ICT group and the SDG group. Empirical analysis was based on data from European statistics on Eurostat and the European Commission.

The study covers the period of 2011–2018, for which we were able to gather the latest statistical data. Not all data showing the impact of the global economic crisis is available, but data have been collected for certain indicators since 2005. We do not have statistical data to show the impact of COVID-19 on the tourism economy (only general studies were available) (WTO 2020). These statistics will be published later.

To monitor the progress towards the Agenda 2030 goals, the European Commission uses 100 different indicators, some of which are not available to all EU countries. This applies, among others, to the indicators describing EU countries with access to the sea in the case of countries that do not have such access. We analyzed all available indicators describing sustainable development and chose typical indicators affecting the tourism industry using an expert study (focus study). These indicators (especially bathing sites with excellent water quality, peaceful and inclusive societies without conflicts or incidents, accountable and inclusive institutions at all levels and climate action) influence the tourist attractiveness of a given country, while respecting the natural environment. However, it should be emphasized that the pursuit of achieving the assumed SDG goals may be tantamount to higher costs. On the other hand, meeting the expectations of this group of tourists who care about respect for the environment. Because we do not examine individual ESG risk factors, they are not grouped into individual factors that reflect environmental, social and economic risks.

Bearing in mind the potential benefits of digitalization on the business environment on the one hand, and the evidence presented by other scholars (Table 1, Table 2 and the critical analysis of the field literature) on the other hand, we proceeded with major research assumptions directing our approach, thus defining three research hypotheses:

#### H1:

 There is a positive relationship between the development of the tourism industry and GDP growth. This means that, in order to achieve a higher level of GDP, a state should plan budgets based on a balanced financial policy. This policy should take into account both the feasibility of revenues as well as sources and methods of spending the funds (expenditure policy). Therefore, it is important to analyze expenditure on digitization.

#### H2:

There is a positive relationship between the level of development of digitization (e-commerce) and the development of the tourism industry.

#### H3:

 There is a positive relationship between the development of the tourism industry and sustainability factors.

### Description of statistical methods

The analyzed features were presented in graphical form as a time series for which basic descriptive statistics were calculated. The upward/downward trend was measured with Kendall’s coefficient for monotonic trend. In this approach, the features are random and the time indicator is deterministic. Finally, bootstrap replicates of a statistic applied to a time series. Phase scrambling is described by Davison and Hinkley (Davison and Hinkley 1999). In our method, 1000 bootstrap replicates of time series are found by taking blocks of length l = 8. The results are given in the form of the T-statistic, Kendall coefficient and *p* value for the independence test.

The correlation between features was given as the Kendall coefficient without bootstrap procedures. The T-statistic and *p* value for the independence test are also provided. The calculations were made in the R language.

Kendall’s Nonparametric Test for Monotonic Trend is a special type of independence test based on Kendall’s statistic. The confidence interval for the slope is computed using modification of the Theil/Sen Method.

In order to select the tools for the study, the test of the variables distribution normality was first performed. Due to the rejection of the hypothesis about the normal distribution of the studied variables at work, non-paramentric tools such as the Kendall's Nonparametric Test were used.

Due to the small number of observations, the conclusions of the trend analysis should be approached with caution. The results show only a tendency, not a clear trend strength.

Annual data were collected from the Eurostat database for the period of 2011–2018. This is due to the fact that, in those years, there was maximum data availability in the field of variables. The variables came from the main tables of the Eurostat database—Data explorer, which is an interface designed for the reading of multi-dimensional tables. All data refer to NACE Rev 2. the nomenclature of economic activities in the European Union (EU). NACE Rev. 2 is to be used, in general, for statistics referring to economic activities performed as from 1 January 2008 onwards. (Eurostat 2008) Data for tourism industry, in a statistical context, refers to the activity of visitors taking a trip to a destination outside their usual environment, for less than a year. It can be for any main purpose, including business or leisure.

We used the following variables for analysis:

- GDP—Gross Domestic Product is an indicator for a nation’s economic situation. It reflects the total value of all goods and services produced minus the value of goods and services used for intermediate consumption in their production. Expressing GDP in PPS (purchasing power standards) eliminates differences in price levels between countries, and calculations on a per-head basis allow for the comparison of economies which are significantly different in terms of absolute size.
- Turnover AFSA—Turnover for accommodation and food service activities comprises the totals invoiced by the observation unit during the reference period, which corresponds to market sales of goods or services supplied to third parties (excluding VAT and other similar deductible taxes directly linked to turnover as well as all duties and taxes on the goods or services invoiced by the unit).
- Access AFSA—Percentage of enterprises which undertake accommodation and food service activities with internet access.
- Web AFSA—Percentage of enterprises which undertake accommodation and food service activities with websites.
- Web order AFSA—Percentage of enterprises which undertake accommodation and food service activities whose websites provide online ordering or reservation or booking, e.g. shopping carts.
- Selling AFSA—Percentage of enterprises which undertake accommodation and food service activities that use online sales (at least 1% of turnover).
- Inland water—The indicator measures the number and proportion of coastal and inland bathing sites with excellent water quality. The indicator assessment is based on microbiological parameters (intestinal enterococci and Escherichia coli). The Bathing Water Directive requires Member States to identify and assess the quality of all inland and marine bathing waters and to classify these waters as ‘poor’, ‘sufficient’, ‘good’ or ‘excellent’.
- Law courts—The indicator measures the total general government expenditure on law courts according to the classification of the functions of government (COFOG). This includes expenditure on administration, operation or support of civil and criminal law courts and the judicial system, including the enforcement of fines and legal settlements imposed by the courts and operation of parole and probation systems; legal representation and advice on behalf of government or on behalf of others provided by government in cash or in services.

### Research results

**Fig. 2:**
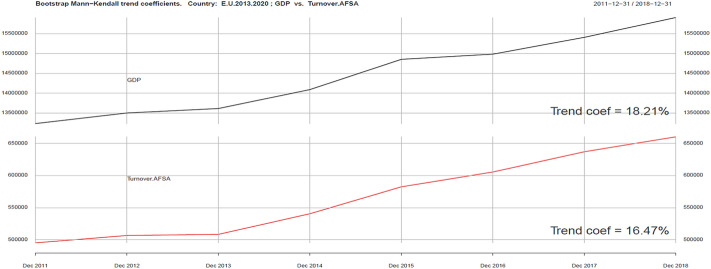
Kendall trend coefficient–EU GDP and EU Turnover AFSA. Source: own elaboration

The first part of the conducted analyses pertained to trends based on Kendall’s trend coefficient. The results—the relationship between the development of the tourism industry and GDP growth in the EU—are presented in Fig. 2.

As we can observe, GDP and Turnover AFSA trend indicators in the EU as a whole are similar (18.21% and 16.27%). The behavior of these variables during the analyzed period (2011–2018) is very similar. The development of the tourism industry and economic growth in EU exhibits very similar trend behavior. We can say that there is a causal relationship between the development of the tourism industry and GDP growth in the EU.

**Fig. 3:**
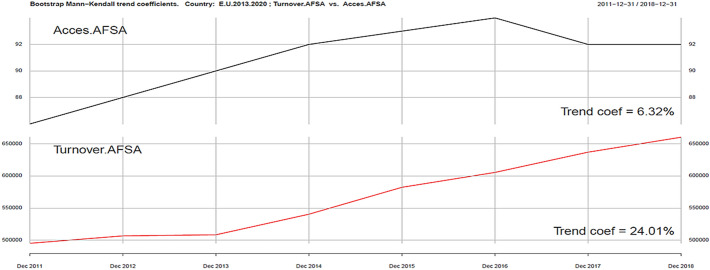
Kendall trend coefficient—EU Access AFSA and EU Turnover AFSA. Source: own elaboration

**Fig. 4:**
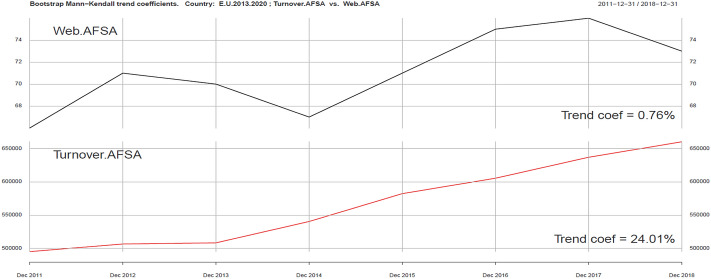
Kendall trend coefficient—EU Web AFSA and EU Turnover AFSA. Source: own elaboration

**Fig. 5:**
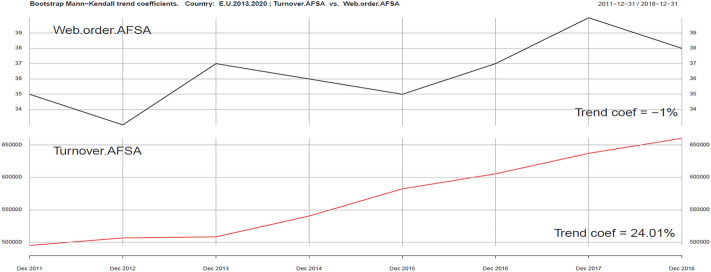
Kendall trend coefficient—EU Web order AFSA and EU Turnover AFSA. Source: own elaboration

**Fig. 6:**
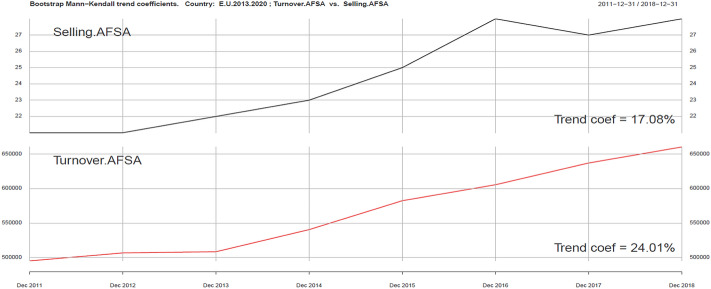
Kendall trend coefficient—EU Selling AFSA and EU Turnover AFSA. Source: own elaboration

Using the same tools, we can analyze ICT and Sustainability development variables in the context of development of the tourism industry (Turnover AFSA). In the ICT group we singled out four variables: Internet Access AFSA, Web AFSA, Web order AFSA and Selling AFSA. They are presented in Figs. 2, 3, 4, 5, 6.

The most similar Kendall’s trend indicator values occur in the case of online sales in the tourism industry (Selling AFSA) and turnover in this part of the economy (Turnover AFSA) (17.08% and 24.01%). A trend less similar to turnover in the tourism industry may be observed in Web order AFSA ( − 1%), Web AFSA (0.76%), and Access AFSA (6.32%). Positive correlation can be seen in the field of ICT variables (excluding Web order). A smaller impact on the level of revenues in the AFSA sector can be clearly seen, in particular in the Web order field, which can be explained by the saturation of ICT tools in the field of online ordering and sales. The use of ICT tools in AFSA is becoming standard.

**Fig. 7:**
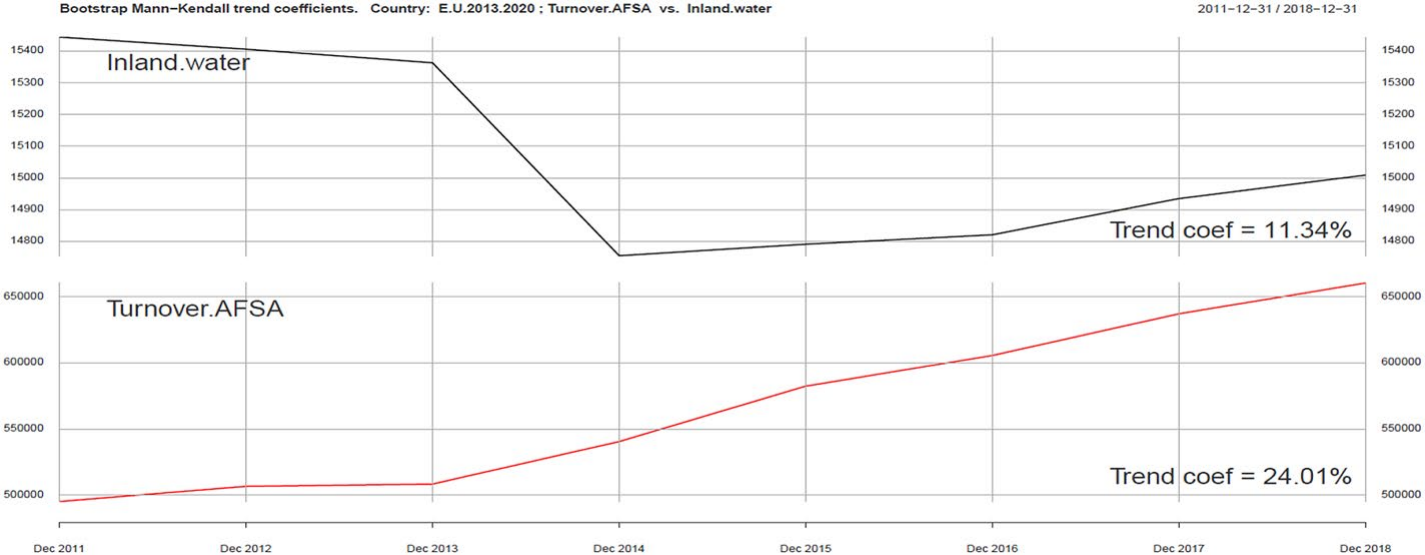
Kendall trend coefficient—EU Inland water and EU Turnover AFSA. Source: own elaboration

**Fig. 8:**
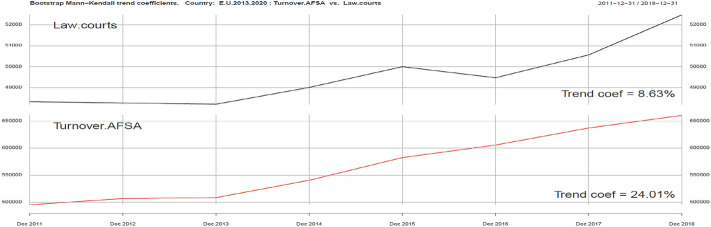
Kendall trend coefficient—EU Law courts and EU Turnover AFSA. Source: own elaboration

The third part of the analysis is connected with sustainability variables: Inland water and Law courts, which are presented in Figs. 7 and 8.

Both of them are only partly correlated with the development of the tourism industry as represented by Turnover AFSA. Kendall’s trend coefficient indicators are 11.34% and 8.63% respectively, while the Turnover AFSA trend indicator is 24.01%. Positive correlation clearly indicates the impact of environmental quality and sustainable development conditions on the level of sales growth in the AFSA sector, especially in terms of security.

The next part of the research analyzed the level of correlation between variables. The correlation statistics for the EU as a whole are presented in Table 3.

**Table 3:** Correlation statistics—EU. Source: own elaboration

Var1	Var2	T-statistic	tau_b Kendalla	*p* value
GDP	Turnover.AFSA	28	1	0
Turnover.AFSA	Inland.water	10	− 0.2857	0.399
Turnover.AFSA	Law.courts	24	0.7143	0.014
Turnover.AFSA	Acces.AFSA	2.2	0.6425	0.03
Turnover.AFSA	Web.AFSA	2.1	0.6183	0.034
Turnover.AFSA	Web.order.AFSA	2	0.593	0.044
Turnover.AFSA	Selling.AFSA	3	0.8895	0.003

The basic correlation between GDP and Turnover AFSA in EU is 1. There is a very strong positive correlation between GDP and the tourism industry (Turnover AFSA). The correlation between the development of the tourism industry and sustainability variables is ambiguous. The Inland water variable and Turnover AFSA are negatively correlated ( − 0.28). On the other hand, the Law courts variable and Turnover AFSA are positively related (0.71).

It is interesting to note that all ICT variables are positively correlated with Turnover AFSA (0.59–0.88). Based on this, we can say that ICT is probably a more important factor in the development of the tourism industry than sustainability variables.

By analyzing GDP and Turnover AFSA in a group of 39 countries, we can find that 15 of them have tau_b Kendall correlation level 1. Another 11 countries have this correlation on a level higher than 0.9. Given that there is a lack of data for four countries, we can say that almost 75% of countries analyzed exhibit a very high correlation between GDP and the development of the tourism industry (Turnover AFSA).

In this context, it is interesting to observe that there is one country (Greece) with negative correlation ( − 0.14). It may be worth analyzing why this highly tourism-oriented country has a negative correlation between GDP and the development of the tourism industry as represented by the Turnover AFSA variable. A very low level of correlation (albeit positive) is also observed in Norway (0.14).

Another part of the analysis relates to the problem of correlation between the development of the tourism industry (Turnover AFSA) and ICT development represented by Web order AFSA. EU countries as a group have a level of correlation of these variables of 0.59. There are countries with high positive correlation: Iceland (1), Denmark (0.89), Hungary (0.70), Sweden (0.69). There are also countries with negative correlation: Austria ( − 1), Cyprus ( − 0.40), Czechia ( − 0.37), Greece ( − 0.37). A third group is comprised of countries with close to zero correlation: Estonia (0), Italy (0.03), Malta (0.03), Norway (0.07), and Germany (0.18). Generally, there are eight countries with negative correlation, nine countries with no data, 20 with positive correlation and 2 with zero correlation. We can assess such findings as indicative of positive correlation between Turnover AFSA and ICT, represented by Web order AFSA.

The final stage of the analysis is connected to sustainable development, represented by Inland water, and the development of the tourism industry, represented by Turnover AFSA. There is a lack of data for 20 countries. 12 countries have positive correlation and six negative. At the EU level, slightly negative correlation ( − 0.28) may be observed.

Countries with higher positive correlation are: Bulgaria (0.84), Spain (0.78) and Croatia (0.71). Higher levels of negative correlation may be observed in Sweden ( − 0.89), Finland ( − 0.87)and Denmark ( − 0.78). The assessment of this phenomenon in the analyzed group of countries is somewhat ambiguous.

## Conclusions and discussion

Existing studies confirm the link between the tourism economy and economic growth. However, research on these matters is still lacking when it comes to a sustainable approach. This paper set out to fill this gap in the literature. Our research has shown that countries in which economic growth is lower (so-called poorer countries) seek to improve the situation by using digitization and emphasizing sustainable development. They attempt to promote the goals of SDGs because, by implementing the idea of sustainability, they see the possibility of using digitization to improve their market position. Thus, they affect sustainable economic development.

Our research has confirmed that there is a relationship between the tourism economy and economic growth and sustainable economic mainstreaming. We can say that there is a causal relationship between the development of the tourism industry and GDP growth in EU. It is interesting to observe that there is one country with negative correlation. It is Greece. It may be worth analyzing why this highly tourism-oriented country has a negative correlation between GDP and the development of the tourism industry. Interesting is also very low level of positive correlation observed in Norway. The correlation between the development of the tourism industry and sustainability variables is ambiguous. Answering the question of whether the digitization of the tourism economy affects the stability of revenues and the functioning of the economy, we can say that ICT is probably a more important factor in the development of the tourism industry than sustainability variables. However, as research shows, ICT tools have had a more significant impact in previous years. At present, it can be concluded that ICT tools have become standard and are no longer such an important factor which influences the development of tourism and increased revenue.

Detailed figures show interesting relationships. It is worth to note that all ICT variables are positively correlated with Turnover AFSA.

Although sustainable development is recognized as an important element affecting the economy and EU countries pay special attention to the sustainability this factor is not taken into account in all countries surveyed (what our research confirmed). In this aspect we can observe that the Inland water variable and Turnover AFSA are slightly negatively correlated. On the other hand, the Law courts variable and Turnover AFSA are positively related. A lack of ambiguity in determining the impact of the tourist economy on sustainable economic growth may be associated with the impact of the effects of the financial crisis, as well as the strong impact of the goals of SDGs through access to EU funds.

Based on this, we can say that ICT is probably a more important factor in the development of the tourism industry than sustainability variables.

Although, as confirmed by the results of the condunducted research, not all analyzed countries show a strong commitment to linking digitization with the goals of sustainable development, it is still necessary to conduct a common policy supporting sustainable development. Through digitization, the society should see how the goals of sustainable development are realized. We make this postulate in connection with the results of our research.

However, without conducting research in this area, it is difficult to obtain an unambiguous answer. The fact is that SDGs have changed over time, and especially in 2020, when—despite the strong impact of digitization on service industries—a significant crisis may observed in the tourism industry in the wake of COVID-19. At the same time, SDGs have changed and were redirected to fight the effects of COVID-19.

Our research attempts to explain the link between sustainability and digitalization in the tourism industry and to contribute to the body of knowledge of factors affecting the tourism industry. The hypothesis (H1.) assuming that there is a positive relationship between the development of the tourism industry and GDP growth has been verified positively. This relationship was presented in Fig. 2 and Table 3. The hypothesis (H2.) assuming that there is a positive relationship between the level of development of digitization (e-commerce) and the development of the tourism industry, and the hypothesis (H3.) assuming that there is a positive relationship between the development of the tourism industry and sustainability factors, have been verified. However, our research shows both negative and positive relationships. For H2. positive relationships are visible for the Scandinavian countries, Iceland and Hungary. Negative relationships occur for Austria ( − 1) and Cyprus ( − 0.40). Hypothesis H3. has been positively verified in most of the surveyed countries, 12 of which have positive trend correlation and six negative. Countries with higher positive correlation are: Bulgaria (0.84), Spain (0.78) and Croatia (0.71). These countries should be considered as countries where the tourism economy belongs to significantly developing industries. But the Scandinavian countries, although they belong to a highly developed economy, they are less attractive to tourists. In addition, Scandinavian countries and Iceland are at the forefront in terms of sustainability Table 4.

**Table 4:** GDP/Turnover AFSA—Correlation statistics. Source: own elaboration

Country	T-statistic	tau_b Kendalla	*p* value
E.U	28	1	0
Austria	28	1	0
Belgium	28	1	0
BosHerz	6	1	0.083
Bulgaria	27	0.9286	0
Croatia	21	0.5	0.109
Cyprus	22	0.5714	0.061
Czechia	27	0.9286	0
Denmark	28	1	0
Estonia	21	1	0
Finland	27	0.9286	0
France	28	1	0
Germany	26	0.8571	0.002
Greece	12	− 0.1429	0.72
Hungary	27	0.9286	0
Iceland	5	0.6667	0.333
Ireland	25	0.7857	0.006
Italy	27	0.9286	0
Kosovo	NA	NA	NA
Latvia	28	1	0
Liechtenstein	NA	NA	NA
Lithuania	28	1	0
Luxembourg	28	1	0
Macedonia	28	1	0
Malta	28	1	0
Montenegro	NA	NA	NA
Netherlands	28	1	0
Norway	16	0.1429	0.72
Poland	27	0.9286	0
Portugal	27	0.9286	0
Romania	27	0.9286	0
Serbia	3	1	0.333
Slovakia	27	0.9286	0
Slovenia	27	0.9286	0
Spain	28	1	0
Sweden	26	0.8571	0.002
Switzerland	26	0.8571	0.002
Turkey	NA	NA	NA
UK	27	0.9286	0

The originality of the research consists of the inclusion of three groups of variables in the analysis (namely the ICT group, the SDG group and the E&T group). This allows the research to contribute to the body of knowledge of factors affecting the tourism industry, in particular providing a new general theory of the influence of sustainability and digitalization on the tourism industry Table 5.

**Table 5:** Turnover AFSA/Web order AFSA—Correlation statistics. Source: own elaboration

Country	T-statistic	tau_b Kendalla	*p* value
E.U	2	0.593	0.044
Austria	0	− 1	0.333
Belgium	NA	NA	NA
BosHerz	NA	NA	NA
Bulgaria	1	0.2965	0.315
Croatia	6	− 0.4286	0.239
Cyprus	− 1.4	− 0.4001	0.17
Czechia	− 1.3	− 0.3706	0.209
Denmark	3	0.8895	0.003
Estonia	0	0	1
Finland	4	0.3333	0.75
France	2.1	0.6183	0.034
Germany	0.6	0.1818	0.533
Greece	− 1.3	− 0.3706	0.209
Hungary	2.3	0.7011	0.023
Iceland	1	1	1
Ireland	18	0.2857	0.399
Italy	0.1	0.0378	0.899
Kosovo	NA	NA	NA
Latvia	0	0	1
Liechtenstein	NA	NA	NA
Lithuania	− 1	− 0.3086	0.304
Luxembourg	15	0.4286	0.239
Macedonia	NA	NA	NA
Malta	0.1	0.0364	0.901
Montenegro	NA	NA	NA
Netherlands	22	0.5714	0.061
Norway	0.3	0.0741	0.802
Poland	2.3	0.6671	0.024
Portugal	0.8	0.2315	0.441
Romania	2.1	0.6183	0.034
Serbia	NA	NA	NA
Slovakia	− 1.6	− 0.4728	0.105
Slovenia	21	0.5	0.109
Spain	1.9	0.5455	0.061
Sweden	2.4	0.691	0.018
Switzerland	NA	NA	NA
Turkey	NA	NA	NA
UK	− 0.9	− 0.2546	0.383

Our recommendations and suggestions are as follows:

1. Governments should take the necessary steps to raise public awareness about relationships between the tourism economy and sustainable economic growth.
2. Tourism economy enterprises should broaden knowledge about the preferences of their customers in the field of sustainability factor. It is about determining whether customers are guided by the criteria of sustainability in making their own decisions regarding the choice of destinations and the environment and conditions of rest.
3. Digitization is not limited by supporting the sustainability factor. Governments and tourism businesses should consider how to establish permanent links between digital and environmental factors to achieve a significant impact on economic growth in the tourism sector.
4. Governments must monitor the tourism economy in terms of the application of green guidelines to build green products dedicated to entities using digitized services.
5. The tourism industry should become familiar with our research and learn which digital and environmental factors have a significant impact on economic growth in the tourism sector.
6. New ICT tools should be developed which incorporate sustainability. This task is addressed to both governments that create intervention programs or support policies and tourism industry enterprises.

Due to the accessibility and comparability of data over time and the specific nature of the phenomenon studied, the authors struggled with a number of limitations during the study. In particular, the selection of variables for the study generated problems. Data on sustainable development dedicated to the tourism economy is very limited Table 6. Within future in-depth research, the authors intend to expand the context of the effect of COVID-19, so as to ensure that ESG risk in the tourism industry is associated with digitization and economic growth. As we have already shown, the impact of the tourism economy on sustainable economic growth is ambiguous, which is why we wish to discern the causes of this ambiguity.

**Table 6:** Turnover AFSA/Inland water—Correlation indicators statistics. Source: own elaboration

Country	T-statistic	tau_b Kendalla	*p* value
E.U	10	− 0.2857	0.399
Austria	NA	NA	NA
Belgium	NA	NA	NA
BosHerz	NA	NA	NA
Bulgaria	2.7	0.8452	0.007
Croatia	24	0.7143	0.014
Cyprus	2	0.6614	0.043
Czechia	NA	NA	NA
Denmark	3	− 0.7857	0.006
Estonia	0.5	0.1782	0.617
Finland	− 2.9	− 0.8693	0.003
France	2.4	0.691	0.018
Germany	− 1.1	− 0.3223	0.294
Greece	− 1.1	− 0.3402	0.254
Hungary	NA	NA	NA
Iceland	NA	NA	NA
Ireland	2.3	0.6671	0.024
Italy	− 0.9	− 0.2646	0.373
Kosovo	NA	NA	NA
Latvia	2	0.6547	0.046
Liechtenstein	NA	NA	NA
Lithuania	NA	NA	NA
Luxembourg	NA	NA	NA
Macedonia	NA	NA	NA
Malta	NA	NA	NA
Montenegro	NA	NA	NA
Netherlands	2.1	0.6172	0.04
Norway	NA	NA	NA
Poland	1.3	0.3706	0.209
Portugal	2.4	0.691	0.018
Romania	NA	NA	NA
Serbia	NA	NA	NA
Slovakia	NA	NA	NA
Slovenia	NA	NA	NA
Spain	25	0.7857	0.006
Sweden	− 3	− 0.8895	0.003
Switzerland	NA	NA	NA
Turkey	NA	NA	NA
UK	1.7	0.4914	0.098

## Footnotes

1 The tourism economy covers the effects of the activities of its various parts. These include accommodation facilities (hotel industry), travel agencies, tourist service offices, tourist transport offices, tourist advertising offices, acquisition and booking offices, youth tourism service units, summer camps, winter camps, training and recreation facilities, qualified tourism facilities and specialist, maritime, aviation, railway, inland, road and mountain units, and institutions producing tourist equipment, footwear, clothing and recreational equipment.
